# Utilization patterns and characteristics of users of biologic anti‐inflammatory agents in a large, US commercially insured population

**DOI:** 10.1002/prp2.708

**Published:** 2020-12-29

**Authors:** Aaron B. Mendelsohn, Young Hee Nam, James Marshall, Cara L. McDermott, Bharati Kochar, Michael D. Kappelman, Jeffrey S. Brown, Catherine M. Lockhart

**Affiliations:** ^1^ Harvard Pilgrim Health Care Institute and Harvard Medical School Boston MA USA; ^2^ AMCP Biologics and Biosimilars Collective Intelligence Consortium Alexandria VA USA; ^3^ Division of Gastroenterology Massachusetts General Hospital Harvard Medical School and Clinical Translational Epidemiology Unit The Mongan Institute Boston MA USA; ^4^ Department of Pediatrics University of North Carolina Chapel Hill NC USA

**Keywords:** anti‐inflammatory, biologics, biosimilars, dermatology, gastroenterology, patterns, rheumatology, treatment, trends, utilization

## Abstract

We report utilization patterns and characteristics of patients treated with biologic anti‐inflammatory agents in a large commercially insured patient population in the United States. We identified adult (age ≥18 years) patients receiving biologic anti‐inflammatory agents between 1 January 2012 and 31 March 2019 across the five Research Partners in the Biologic and Biosimilars Collective Intelligence Consortium's Distributed Research Network. We examined the number of incident use episodes for each biologic, as well as patient demographic and clinical characteristics. Curated data and analytic tools from the Food and Drug Administration's Sentinel System were used to perform the analyses. We identified 90,360 incident episodes of tumor necrosis factor‐alpha inhibitors (TNFi) and 70,506 incident episodes of non‐TNFi medications. Adalimumab was the most common TNFi drug (47% of all TNFi episodes) and showed a steady increase in utilization during the study period compared to other TNFi agents. Rituximab was the most commonly initiated non‐TNFi medication (44% of non‐TNFi episodes). Other non‐TNFi agents, namely, ustekinumab, vedolizumab, and secukinumab, demonstrated notable increases in utilization over time. Biosimilar use was limited; we observed 653 incident episodes for infliximab‐dyyb and 39 incident episodes for infliximab‐abda. As more biologics enter the market, greater variation in the use of biologics with similar indications and between biologic originators and biosimilars is anticipated. Because information on efficacy and safety at the time of drug approval is limited, post‐marketing surveillance and research is needed to monitor medication safety and evaluate effectiveness between biologic drugs using real‐world data.

**Abbreviations**
BBCIC: Biologics and Biosimilars Collective Intelligence Consortium
DRN: distributed research network
RA: rheumatoid arthritis
TNFi: tumor necrosis factor‐alpha inhibitor

**Key Points**

- We analyzed utilization trends and patient characteristics for biologic anti‐inflammatory drugs available in the United States using a large healthcare claims database (Biologic and Biosimilars Collective Intelligence Consortium's Distributed Research Network) from January 2012 to March 2019.
- The total number of incident utilization episodes of biologic anti‐inflammatory agents steadily increased from 2012 to 2017 (the most recent year of complete data).
- Adalimumab and rituximab were the most commonly initiated tumor necrosis factor‐alpha inhibitor (TNFi) and non‐TNFi agent, respectively.

## 1 INTRODUCTION

Since the first biologic agent, recombinant human insulin, was approved in 1982, biologics have accounted for one of the fastest growing segments of the prescription drug market in the United States (US).^1^, ^2^ Biologic anti‐inflammatory agents are used for the treatment of various, and oftentimes multiple, inflammatory diseases, including, but not limited to, rheumatoid arthritis (RA), psoriasis, Crohn's disease, ulcerative colitis, and multiple sclerosis.^3^ Compared to chemically synthesized small‐molecule drugs, large molecule biological medications are structurally more complex, and have the potential to be more targeted therapies. They also have limitations such as efficacy and safety concerns related to immunogenicity, and inherent natural variability.^4^, ^5^ Furthermore, the introduction of biosimilars has introduced even more complexity and opportunity into the real‐world assessment of biologics for the treatment of anti‐inflammatory disease.

This Short Report presents usage patterns and characteristics of patients treated with biologic anti‐inflammatory agents in a large distributed network of commercially insured patients in the United States. Whereas previous utilization studies have generally focused on single or selected biologic agents (e.g., tumor necrosis factor (TNF)‐alpha inhibitors (TNFi) only) and have typically not considered indications across all therapeutic areas, our descriptive report provides a broad perspective of all relevant agents, indications, and all therapeutic areas.

## 2 MATERIALS AND METHODS

We used data from the distributed research network (DRN) of the Biologics and Biosimilars Collective Intelligence Consortium (BBCIC), a non‐profit research consortium dedicated to investigation of the real‐world effectiveness and safety of biologics, including biosimilars in the US (www.bbcic.org). The BBCIC DRN consists of administrative claims data for approximately 95 million patient‐years from five Research Partners including two large national health insurers (Healthagen [Aetna], HealthCore [Anthem]) and three regional health insurers or integrated healthcare delivery systems (Harvard Pilgrim Health Care, HealthPartners, Kaiser Permanente of Washington). These Research Partners participate in the Food and Drug Administration's (FDA) Sentinel System, enabling the BBCIC to leverage the curated data in the Sentinel Common Data Model, plus data standardization and analytic tools of the Sentinel System for conducting distributed analyses.^6^, ^7^, ^8^

We identified adult (age ≥18 years) patients receiving biologic anti‐inflammatory agents between 1 January 2012 and 31 March 2019 in the BBCIC DRN; some Research Partners provided data through only part of 2018. Medications of interest were currently available anti‐inflammatory biologic drugs in the US including both TNFi and non‐TNF‐alpha inhibitors (non‐TNFi); a full list of medications is provided in Table 1. Medications were identified using J‐codes from Healthcare Common Procedure Coding System and National Drug Codes. The complete list of these codes can be found at the BBCIC website (https://www.bbcic.org/research/completed‐research).

**TABLE 1:** Patient characteristics of incident episodes of biologic anti‐inflammatory agents in the Biologics and Biosimilars Collective Intelligence Consortium Distributed Research Network, 1 January 2012 – 31 March 2019

A. TNF‐alpha inhibitors	adalimumab	certolizumab	etanercept	golimumab	infliximab
Number of incident episodes	42,622	5,303	21,028	5,526	15,189
Demographic characteristics					
Mean (SD) age in years	46.8 (13.8)	51.0 (15.6)	50.0 (12.7)	52.3 (14.6)	47.8 (16.4)
Age groups, number (%)					
18–49 years	23,355 (54.8)	2,399 (45.2)	9,484 (45.1)	2,226 (40.3)	8,067 (53.1)
50–64 years	16,334 (38.3)	1,887 (35.6)	9,706 (46.2)	2,308 (41.8)	4,755 (31.3)
65+ years	2,933 (6.9)	1,017 (19.2)	1,838 (8.7)	992 (18.0)	2,367 (15.6)
Female, number (%)	24,235 (58.0)	3,760 (71.8)	13,284 (64.7)	3,880 (71.1)	8,867 (59.3)
Clinical characteristics, number (%), unless otherwise noted					
Charlson/Elixhauser Combined Comorbidity Score, mean (SD)	0.5 (1.2)	0.6 (1.3)	0.4 (1.1)	0.6 (1.3)	0.9 (1.7)
Rheumatoid arthritis	16,672 (39.1)	3,408 (64.3)	14,061 (66.9)	4,179 (75.6)	5,548 (36.5)
Inflammatory bowel disease	11,898 (27.9)	1,566 (29.5)	500 (2.4)	888 (16.1%)	8,704 (57.3)
Psoriasis, psoriatic arthritis, ankylosing spondylitis	18,987 (44.5)	1,886 (35.6)	9,707 (46.2)	1,909 (34.5)	3,343 (22.0)
Sjögren's syndrome	735 (1.7)	154 (2.9)	597 (2.8)	169 (3.1)	242 (1.6)
Systemic lupus erythematosus	519 (1.2)	109 (2.1)	449 (2.1)	136 (2.5)	214 (1.4)

	infliximab‐abda	infliximab‐dyyb
Number of incident episodes	39	653
Demographic characteristics		
Mean (SD) age in years	53.8 (16.9)	54.2 (17.6)
Age groups, number (%)		
18–49 years	>10 (NC)	259 (39.7)
50–64 years	<10 (NC)	191 (29.2)
65+ years	>10 (NC)	203 (31.1)
Female, number (%)	26 (66.7)	368 (56.5)
Clinical characteristics, number (%), unless otherwise noted		
Charlson/Elixhauser Combined Comorbidity Score, mean (SD)	1.0 (1.4)	0.9 (1.7)
Rheumatoid arthritis	18 (46.2)	305 (46.7)
Inflammatory bowel disease	20 (51.3)	323 (49.5)
Psoriasis, psoriatic arthritis, ankylosing spondylitis	<10 (NC)	167 (25.6)
Sjögren's syndrome	<10 (NC)	13 (2.0)
Systemic lupus erythematosus	0 (0.0)	<10 (NC)

B. Non‐TNF‐alpha inhibitors	abatacept	anakinra	brodalumab	canakinumab	guselkumab
Number of incident episodes	7,745	289	67	82	976
Demographic characteristics					
Mean (SD) age in years	56.2 (13.2)	48.7 (15.6)	51.5 (11.3)	41.3 (15.0)	49.5 (12.8)
Age groups, number (%)					
18–49 years	2,240 (28.9)	150 (51.9)	>10 (NC)	>10 (NC)	482 (49.4)
50–64 years	3,709 (47.9)	97 (33.6)	>10 (NC)	>10 (NC)	403 (41.3)
65+ years	1,796 (23.2)	42 (14.5)	<10 (NC)	<10 (NC)	91 (9.3)
Female, number (%)	6,123 (80.8)	166 (58.9)	34 (50.7)	52 (63.4)	463 (47.4)
Clinical characteristics, number (%), unless otherwise noted					
Charlson/Elixhauser Combined Comorbidity Score, mean (SD)	0.7 (1.6)	1.9 (2.5)	0.3 (1.1)	1.0 (2.0)	0.3 (1.2)
Rheumatoid arthritis	7,388 (95.4)	212 (73.4)	<10 (NC)	49 (59.8)	70 (7.2)
Inflammatory bowel disease	235 (3.0)	19 (6.6)	<10 (NC)	<10 (NC)	28 (2.9)
Psoriasis, psoriatic arthritis, ankylosing spondylitis	1,169 (15.1)	25 (8.7)	66 (98.5)	<10 (NC)	969 (99.3)
Sjögren's syndrome	468 (6.0)	<10 (NC)	0 (0.0)	<10 (NC)	<10 (NC)
Systemic lupus erythematosus	419 (5.4)	24 (8.3)	<10 (NC)	<10 (NC)	<10 (NC)

	ixekizumab	natalizumab	rilonacept	rituximab	sarilumab
Number of incident episodes	1,856	2,788	22	31,371	305
Demographic characteristics					
Mean (SD) age in years	50.1 (11.7)	44.3 (11.6)	49.4 (20.4)	62.3 (15.0)	54.8 (11.6)
Age groups, number (%)					
18–49 years	886 (47.7)	1,904 (68.3)	>10 (NC)	5,969 (19.0)	96 (31.5)
50–64 years	827 (44.6)	781 (28.0)	<10 (NC)	11,750 (37.5)	168 (55.1)
65+ years	143 (7.7)	103 (3.7)	<10 (NC)	13,652 (43.5)	41 (13.4)
Female, number (%)	894 (48.2)	1,989 (73.0)	<10 (NC)	14,744 (50.8)	249 (81.6)
Clinical characteristics, number (%), unless otherwise noted					
Charlson/Elixhauser Combined Comorbidity Score, mean (SD)	0.3 (1.2)	0.5 (1.2)	0.5 (0.8)	2.8 (2.9)	0.8 (1.5)
Rheumatoid arthritis	237 (12.8)	99 (3.6)	<10 (NC)	6,197 (19.8)	303 (99.3)
Inflammatory bowel disease	36 (1.9)	108 (3.9)	0 (0.0)	981 (3.1)	<10 (NC)
Psoriasis, psoriatic arthritis, ankylosing spondylitis	1,834 (98.8)	74 (2.7)	<10 (NC)	1,651 (5.3)	35 (11.5)
Sjögrens disease	<10 (NC)	13 (0.5)	<10 (NC)	803 (2.6)	28 (9.2)
Systemic lupus erythematosus	14 (0.8)	14 (0.5)	<10 (NC)	1,179 (3.8)	11 (3.6)

	secukinumab	tocilizumab	ustekinumab	vedolizumab
Number of incident episodes	5,398	4,670	10,208	4,729
Demographic characteristics				
Mean (SD) age in years	50.0 (12.0)	56.3 (13.7)	47.4 (13.9)	44.5 (15.4)
Age groups, number (%)				
18–49 years	2,513 (46.6)	1,342 (28.7)	5,617 (55.0)	3,003 (63.5)
50–64 years	2,469 (45.7)	2,198 (47.1)	3,729 (36.5)	1,268 (26.8)
65+ years	416 (7.7)	1,130 (24.2)	862 (8.4)	458 (9.7)
Female, number (%)	2,817 (52.4)	3,629 (78.9)	5,051 (50.6)	2,430 (51.8)
Clinical characteristics, number (%), unless otherwise noted				
Charlson/Elixhauser Combined Comorbidity Score, mean (SD)	0.4 (1.2)	0.7 (1.6)	0.5 (1.3)	1.1 (1.7)
Rheumatoid arthritis	1,461 (27.1)	4,416 (94.6)	1,454 (14.2)	373 (7.9)
Inflammatory bowel disease	138 (2.6)	150 (3.2)	2,258 (22.1)	4,694 (99.3)
Psoriasis, psoriatic arthritis, ankylosing spondylitis	5,297 (98.1)	598 (12.8)	8,358 (81.9)	435 (9.2)
Sjögrens disease	56 (1.0)	259 (5.5)	46 (0.5)	13 (0.3)
Systemic lupus erythematosus	53 (1.0)	191 (4.1)	84 (0.8)	39 (0.8)

Notes

‐ NC: Not Calculated. Small number of counts (0 < n < 10) are suppressed (indicated as <10 in the table), and their proportions are not calculated. Cells that allow a value of 1 to 9 to be derived are also suppressed. SD: standard deviation. TNF: tumor necrosis factor.

Tildrakizumab‐asmn was not included in the analyses as there were <10 episodes for this product.

Data from 1 January 2012 to as recent as 31 March 2019 were analyzed. Some Research Partners provided data through part of 2018.

Data are based on incident episodes, not unique users, except for the data stratified by sex that are based on unique users. The number of incident episodes may not be the same as the number of unique users. Individuals were counted multiple times if inclusion/exclusion criteria for incident episodes were met.

Sum of the numbers within a category may not be 100.0% because of rounding.

All patients were required to be continuously enrolled with medical and pharmacy coverage for a minimum of 365 days before the first observed administration/dispensing date for a medication of interest (index date). Coverage gaps of up to 45 days were bridged to account for potential administrative lapses in coverage. Incident use was defined as having no exposure to a given medication in the 365 days before the index date. Patients could contribute multiple “incident” episodes for the same or different drugs if they met the eligibility criteria, including the 365‐day washout period, each time. We examined the number of incident use episodes for each medication of interest, overall and according to patient demographic and clinical characteristics (i.e., comorbidity scores and common indications for anti‐inflammatory medications). Demographic characteristics were measured on the index date, and clinical characteristics were assessed during the 183‐day period before the index date (“baseline period”). The publicly available Sentinel System analytic toolkit (Cohort Identification and Descriptive Analysis [CIDA v8.1.0]) was used to perform the distributed analyses.^9^

Distributed analyses were conducted by each Research Partner, with results aggregated for final analysis. Cells with counts >0 and <10 are not presented to protect the anonymity of patients, per standard BBCIC practice; medications with <10 incident episodes are excluded from analysis. Institutional Review Boards for the participating Research Partners determined that this work does not meet the definition of human subjects research.

## 3 RESULTS

Over 30 million eligible health plan members who contributed more than 75 million person‐years met the study eligibility criteria. We identified 160,866 incident episodes across all study medications, including 90,360 incident episodes of TNFi products and 70,506 incident episodes for non‐TNFi medications (Table 1). We did not include tildrakizumab‐asmn as there were <10 incident episodes for this medication. Adalimumab was the most common TNFi with 42,622 incident episodes (47.2% of all TNFi episodes), followed by etanercept (n = 21,028, 23.3%) and infliximab (n = 15,189, 16.8%). Rituximab was the most commonly initiated non‐TNFi medication with 31,371 episodes (44.5%) of all non‐TNFi episodes, followed by ustekinumab (n = 10,208, 14.5%) and abatacept (n = 7,745, 11.0%). We observed utilization for a biosimilar, infliximab‐dyyb, beginning in 2017 with 92 episodes, and increasing to a total of 634 episodes by 2018. Thirty‐nine (39) episodes were observed for a second infliximab biosimilar, infliximab‐abda, beginning in 2018.

As shown in Table 1, the mean ages of patients ranged from 41 (canakinumab) to 62 years (rituximab) and most users across medications were female (all except ixekizumab and guselkumab). The average Charlson/Elixhauser Comorbidity Score^10^ ranged from 0.3 (brodalumab, guselkumab, ixekizumab) to 2.8 (rituximab). The proportions of episodes by conditions were consistent with the labeled indications for these medications. For example, 95% of the incident abatacept episodes included a previous diagnosis of RA; and, for adalimumab with multiple indications across therapeutic areas, 39%, 28%, and 45% of the episodes included a previous diagnosis of RA, inflammatory bowel disease, and psoriasis/psoriatic arthritis/ankylosing spondylitis, respectively.

**FIGURE 1:**
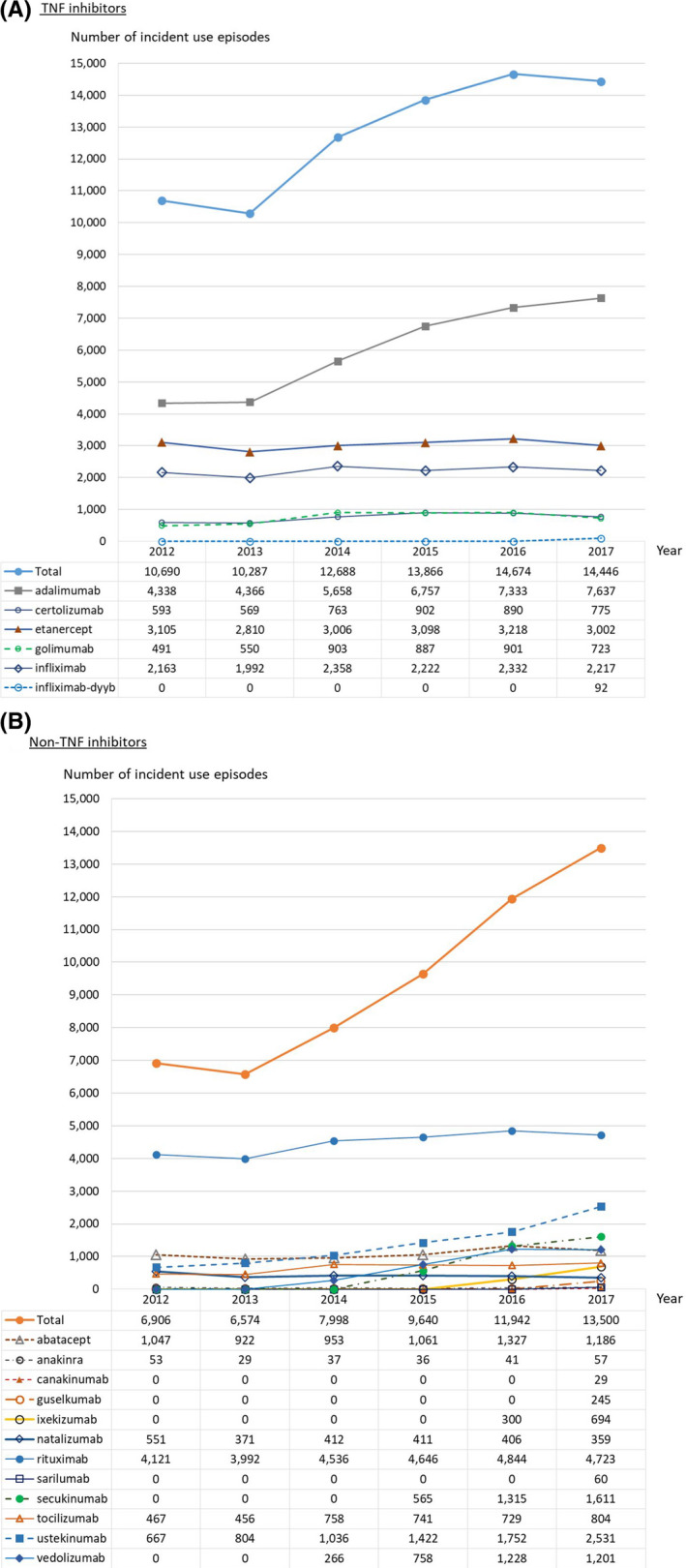
Number of incident use episodes of biologic anti‐inflammatory agents by year in the Biologics and Biosimilars Collective Intelligence Consortium Distributed Research Network, 01 January 2012—31 December 2017

Figure 1 presents trends in the number of incident drug episodes by year from 2012 to 2017 (as 2018 and 2019 data were partially complete, these years are not included in the figure). Adalimumab showed steady increase over time, from 4,338 incident episodes in 2012 to 7,637 in 2017. Other TNFi agents remained relatively stable over time. Use of rituximab did not appear to substantially change over the study period, however, incident episodes of other non‐TNFi products, including ustekinumab, and newer agents, namely vedolizumab and secukinumab, demonstrated increasing utilization over time.

## 4 DISCUSSION

In this longitudinal utilization study, we examined the number of incident use episodes and patient characteristics for users treated with biologic anti‐inflammatory agents in the BBCIC DRN from 2012 to 2019. The results show that adalimumab and rituximab were the medications with the largest number of incident episodes among TNFi and non‐TNFi agents, respectively. Also, adalimumab had the highest increase in the number of incident episodes (through 2017, the most recent year of complete data), while the utilization for rituximab was relatively stable.

The biologic agents with more than 10,000 incident use episodes were adalimumab, rituximab, etanercept, infliximab, and ustekinumab. All of these drugs were initially approved by the FDA before 2010, had expanded indications over time, and included indications across therapeutic areas. The biosimilar category included two drugs, infliximab‐adba (approved in 2017) and infliximab‐dyyb (approved in 2016). As these biosimilars are relatively new, it is still early to evaluate the trends of their utilization and the potential impact on the originator drug. The implications of biosimilars in the anti‐inflammatory biologics space in the US remain uncertain until further data on utilization become available.

Several factors need to be considered in interpreting these results. Our data are generally representative of commercially insured individuals in the United States. Whether these findings would be applicable to other populations, for example, older patients with only Medicare insurance coverage, is uncertain. Also, as 2018 and 2019 data were partially complete, we were not able to fully analyze trends in utilization beyond 2017. Finally, there is the possibility of misclassification in identifying drug exposures and clinical characteristics through administrative claims data.

As more biologics and biosimilars enter the market, it is anticipated that there will be a greater variation in the use across biologic products and more competition among products with similar indications and between biologic originators and biosimilars. Because information on rare but potentially serious adverse events at the time of drug approval is limited, post‐marketing surveillance will be important to monitor adverse drug events and evaluate outcomes between biologics and biosimilars in the real‐world environment. Comparative effectiveness and safety studies among these drugs are also important to inform clinical decisions for optimal treatment choices customized for individual patients. Given that biologic agents can be used for a variety of indications and patients may switch drugs, studies on indication‐specific benefits/risks of biologics and the impact of switching biologic agents on the treatment effectiveness and safety are warranted. Also, further research on potential factors associated with utilization patterns will be informative, including cost of treatments, route of administration, prevalence of indications, adverse drug events, step therapy, patient and provider preferences, and pharmacy benefit programs. Ongoing monitoring of biologics utilization patterns will help strategically identify future research priorities.

## 5 ETHICS APPROVAL

Institutional Review Boards for the participating Research Partners determined that this work does not meet the definition of human subjects research.

## CONFLICT OF INTEREST

Michael Kappelman is a consultant to Janssen, AbbVie and Takeda and receives research support from Janssen and AbbVie. All other authors have no competing interests.

## AUTHOR CONTRIBUTIONS

All authors contributed to the study conception and design for this work. Data analysis was led by James Marshall with input and direction from all other authors. Aaron Mendelsohn and Young Hee Nam prepared the first draft of the manuscript and all authors participated in the revision of the initial manuscript, and approved the final manuscript. All authors agree to be accountable for all aspects of the work.

## Data Availability

The data analyzed for this study are available from the corresponding author on reasonable request.
